# Socially Inclusive Development: The Foundations for Decent Societies in East and Southern Africa

**DOI:** 10.1007/s11482-016-9491-6

**Published:** 2016-11-07

**Authors:** Pamela Abbott, Claire Wallace, Roger Sapsford

**Affiliations:** School of Social Sciences, University of Aberdeen, Aberdeen, UK

**Keywords:** Social inclusion, Inclusive development, East Africa, Sub-Saharan Africa, Decent society

## Abstract

This article is concerned with how social processes and social provision are conceptualised and measured in societies in order to offer guidance on how to improve developmental progress. Significant advances have been made in developing multidimensional measures of development, but they provide little guidance to governments on how to build sustainable societies. We argue for the need to develop a theoretically informed social and policy framework that permits the foundations for building decent societies to be put in place by governments. In our view the recently developed Decent Society Model provides such a framework. Our example is the assessment of government provision, by function, within fourteen countries of East and Southern Africa. The context is the current debates about socially inclusive development, but we argue that it is necessary to range more widely, as social processes of different kinds are multiply interrelated. Social inclusion is recognised by governments as well as international agencies, including the World Bank and the United Nations, as not only an ethical imperative but smart economics; socially inclusive societies are more stable and have greater potential for economic growth. Societies that can develop sustainably need not only to be inclusive, however, but to provide economic security for all, to be socially cohesive and to empower citizens so that as individuals and communities they can take control over their own lives.

## Introduction

The focus of this article is how societies need to understand themselves - what conceptual framework should be used - in order to improve the quality of life for their residents. This is not an ‘academic’ issue in the pejorative sense of being a way for outsiders to classify and analyse specimens. On the contrary, it is an intensely practical matter; for effective action, governments need a ‘theory of society’ to direct their policies and practices and a set of statistical and analytical tools to measure the size of problems, identify priority targets and evaluate performance. However, successful governance does not depend just on the manipulation of measures or qualities; it requires looking at the society as a whole rather than a sum of parts and conceptualising it in terms of processes rather than conditions or milestones. Any competent government manipulates the socioeconomic situation of a country using some theoretical perspective and follows a (perhaps only implicitly) theorised understanding of what makes societies work, in order to provide resources and incentives and empower people to change their own lives rather than be changed by others. (This is a different question from whether they govern in an authoritarian manner, though obviously related to it; even the most ‘democratic’ of governments manipulates the country’s situation as an inextricable part of governing.) Governments therefore need to know what provision might improve the society in desired ways, more than they need to know what they have already achieved.

To talk sensibly about this at finite length we have had to limit what the article covers. It concentrates on fourteen countries of East and South Africa for which the information which we require is available. They show sufficiently substantial problems to make social change a necessity for them and sufficient variation to demonstrate both difference and similarity. They exemplify different approaches to government, and some of them have a governmental style which implicitly draws on the model that we are advocating here, characterising social processes and what needs to be done to make a decent society in similar ways to ours. We have not tried to tackle every aspect of the complex social whole in this article but focused mostly on the concept of ‘inclusive development’, by which is meant development which benefits all residents, not just an elite but also not exclusively dominated by ‘pro-poor’ policies which benefit only those at the bottom of the ladder. We try to keep in mind, however, that processes of inclusion are only part of the complex of social relations and should not be regarded as whole answer.

This article offers a brief review of well-known approaches to how states are performing, each of which embodies an ontology (i.e. defines what the important features are in the situation). It goes on to apply a development out of the existing literature on Social Quality – the Decent Society model (Abbott et al. 2016) and its associated Decent Society Index – which has the potential to capture the complex and holistic interaction of social processes within the fourteen African countries a little more clearly and to provide a framework within which changes could be tried out and assessed.

## Social Inclusion

Focusing on social inclusion raises the question of *inclusion in what*: in what type of society are people to be included? It requires us to ask in what type of society we want to live and who the ‘we’ are that answer that question and are to be included. We need to consider what the values of an inclusive society are and what the institutional arrangements that would embody these would be. Social inclusion is a process that aims to create a ‘society for all’, a society in which no one is left behind. It is about guaranteeing human rights and promoting social justice for all, increasing the quality of life of citizens and improving individual wellbeing. There are economic (mainly monetary and financial) issues, social issues (social relationships with family, friends, neighbours, local communities, social activism, participation in civil society) and political/institutional issues (relationship between individuals/groups and the state, including participation in decision making, access to information and an independent judicial system and respect for the rule of law); these are all dimensions of social inclusion. Creating a society for all is a moral obligation, a commitment to upholding the human rights of everyone and creating just societies, but there are also strong instrumental reasons for promoting social inclusion. Socially inclusive societies are safer and more stable, and they meet the essential conditions for economic transformation and growth, high levels of productive employment and social cohesion (Abbott et al. 2016; DESA 2009; World Bank 2013).

An important issue is the inclusion of the poor, with poverty being seen as a barrier to full participation. Social inclusion is about more than poverty reduction and pro-poor economic policies, however; it is about enabling all to benefit from economic growth – inclusive development as well as pro-poor development (African Union 2008a, b; Rahim 2009). The challenge is to understand what precisely governments can do to promote social inclusion. Policies and legal frameworks have to be developed and implemented that not only promote social inclusion but also guarantee citizens economic security and empower them within a socially cohesive society. The goal of social inclusion can be achieved only if citizens have the capability to participate (are empowered), the financial resources to take part in the taken-for-granted daily activities of the society in which they live (economic security) and a social order where competing interest-groups are able to respect difference and work together, following agreed ‘rules of the game’ (social cohesion). This vision of a decent society is encapsulated in the Social Policy Framework for Africa (African Union 2008b; African Union Commission 2014) and the UN vision of social integration (DESA 2009). Achieving the Sustainable Development Goals will require transforming societies so that they strive to become ‘decent societies’ (Abbott et al. 2016; World Bank 2013).

A fully inclusive society can be one in which individuals, groups and communities participate in social interaction without necessarily requiring that every citizen plays an active role (Therborn 2007) while ensuring that individuals and groups have access to the resources necessary to take up opportunities that enable them to live a fulfilling life (Sen 1999). The five dimensions of inclusion, from lowest to highest are: *visibility* - to be recognised as a member of the society; *consideration* - that the needs and concerns of all individuals and groups in society are taken into account by policy; *access to social interactions* - that everyone has the same rights to participate; *equal rights* - that the human rights set out in international law are domesticated and all members of society are able to claim them; and *access for all to resources* necessary to participate fully in society (Therborn, quoted in DESA 2009). This five-part goal has not been fully achieved in any country in the world; it is an ideal, something towards which countries can attempt to move, and all countries are ‘works in progress’ with respect to it.

While the initial emphasis of world social policy has been on economic inclusion through poverty reduction, social protection and employment creation, it has increasingly become recognised that social inclusion has social and political dimensions as well as economic ones and that it is about more than income and employment. It is about the capacity of people to participate in their society, and lack of financial resources is only one barrier to full participation. An inclusive society is one where all can achieve their full potential regardless of individual circumstances, constrained only by what is needed for others being able to exercise the same rights and have access to the same opportunities.

An inclusive society is one that rises above differences of race, gender, class, generation and geography to ensure equality of opportunity regardless of origin, and one that subordinates military and economic power to civil authority. In an inclusive society, social interaction is governed by an agreed set of social institutions. The capability of all citizens to determine how those institutions function is indeed a hallmark of an inclusive society. (Atkinson and Marlier 2010, p. 2).

Social inclusion is one of the key challenges facing countries in East and Southern Africa and across the world (see e.g.: Abbott et al. 2014; DESA 2009; Semkwiji 2009); it is a necessity for the creation of safe, stable and just societies. Social inclusion is both a process and an outcome. As a process it ensures that poor and excluded individuals and communities gain the opportunities and resources necessary to participate fully in economic, social and cultural life and to enjoy the standard of living and well-being that is considered normal in the society in which they live. It also ensures that they have a say in decisions which affect their lives and access to their fundamental rights. Thus it involves redistribution, recognition and participation in decision making - see Fraser (2010). Social inclusion is relative to a given society and a multidimensional and complex concept which recognises that marginalisation and exclusion is about more than poverty. The poor are not one homogeneous group, and non-poor individuals can also be socially excluded because of stigmatised and devalued characteristics (and individuals and groups can be marginalised and excluded because of more than one characteristic). Grounds for intersectional or compounded discrimination may include gender, ethnicity/race, indigenous or minority status, colour, socio-economic status and/or caste, language, religion or belief, political opinion, national origin, marital and/or maternal status, age, urban/rural location, health status, disability, property ownership and being lesbian, bisexual or transgender or intersex persons. Marginalised and excluded groups are often minorities, but this does not have to be the case; women make up half the population but are politically marginalised in most states, and elite capture of the state can mean that a minority dominate a majority, as happened in South Africa under apartheid.

## Models of Governance for Evaluating Development

### The State as an Economic System

**Fig. 1:**
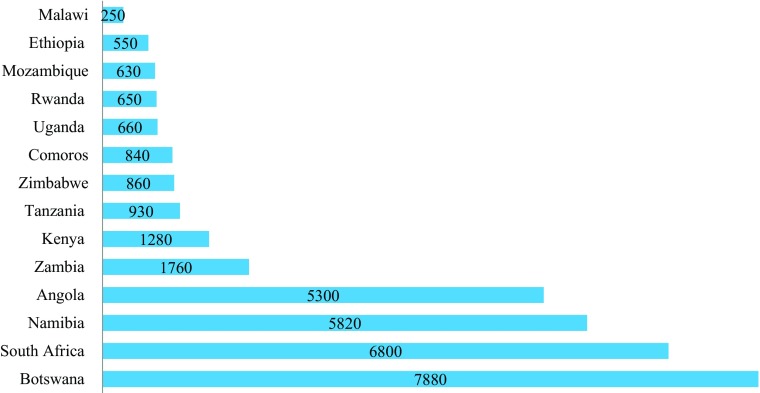
GNI per Capita Atlas Method US$ (PPP), 2014, for Selected East and Southern African Countries. Source: data from World Bank - World Development Indicators

The main way in which socioeconomic progress has been measured (and it still is) is by using economic indicators, with the dominant one being Gross Domestic Product (GDP) or Gross National Income (GNI), generally reported per capita. Figure 1 shows the GNI per capita for fourteen countries in East and Southern Africa (the ones for which Decent Society Index scores are available). There is significant variation in the progress of the fourteen countries based on this measure; the poorest countries are among the poorest in the world, while the better-off countries fall in the ‘upper middle income’ category. The seven poorest countries are all categorised as ‘least developed countries’, ones that are recognised as facing especial difficulties in moving onto a path of sustainable development. Comoros is, additionally, recognised as especially disadvantaged because it is a small island state and Malawi, Rwanda, Uganda, Zambia and Zimbabwe because they are landlocked less developed states.

One problem with GDP and GNI is that they tells us nothing about how the wealth of a country is distributed, and an increase in GNI per capita may not bring equal benefit to all citizens; economic inequalities, for example, may not change or may even increase as a country’s economy grows. It also tells us nothing about the extent to which the quality of life is improving. There is a danger that economic growth will become the main objective rather than being seen as a means to increasing citizens’ quality of life. Thus policies that focus just on economic growth may not move countries onto a path of sustainable development and economic growth may itself be held back by a lack of social integration, social inclusion and social empowerment (Abbott et al. 2016; DESA 2009; Stiglitz et al. 2009; World Bank 2013).

**Fig. 2:**
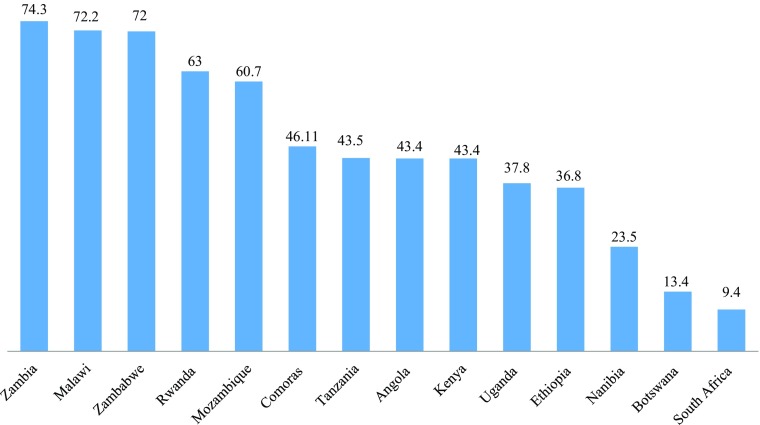
Population Living on Less than US$ 1.25 ppp a Day, 2012 or Nearest Year, for Selected East and Southern African Countries (%) Source: data from World Bank - World Development Indicators

There are a number of alternative measures of the economic situation of a country as it affects the quality of life of citizens. The most frequently used is poverty, with absolute poverty defined as having less than US$ 1.25 ppp a day (Fig. 2). While there is an obvious correlation between poverty and GNI per capita, nevertheless some countries seem better than others at economic inclusion. Zambia, for example has the highest poverty rate but a higher GNI per capita than seven countries that have lower poverty rates. Zimbabwe, Angola and Botswana also have higher poverty rates than other countries with lower GNI per capita. By contrast, Rwanda, Comoros, Mozambique and South Africa have lower rates of poverty than might be expected from their GNI relative to other countries.

There has been a move over the last 20 or so years from using GDP as the sole or main measure of social progress to multidimensional measures. This is an important development, but if it is tied to a fundamentally economic understanding of state and governance it remains insufficient to provide governments with sufficient guidance for building inclusive, decent societies.

### Bringing in the Social

The question of how to measure socioeconomic progress is hotly contested, and over the last 10 to 20 years policy makers have increasingly come to accept that measuring progress by the extent to which a country is becoming better off is not adequate - that for inclusive and sustainable development it is important to take account of indicators of people’s quality of life and wellbeing. However, there continues to be debate about which the most informative measures of progress are, on the one hand between those that argue for a single index or indicator and those that argue for a dashboard, and on the other between those that argue for objective measures of a country’s or its residents’ progress and those that argue that people’s views should at least be taken into account. An increasing number of composite indexes and dashboard are being developed which respond to criticisms that there has been too narrow a focus on economic factors by aiming to measure more broadly the quality of life people are able to access.

**Fig. 3:**
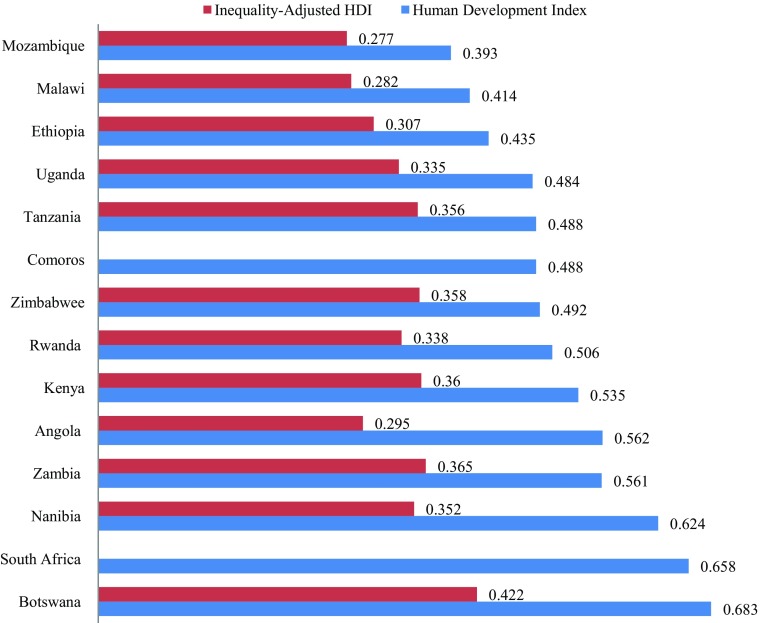
UNDP 2014 Human Development Index and Inequalities-Adjusted Human Development Index for Selected East and Southern African Countries Source: UNDP - Human Development Indicators

Probably the best known of these is the UNDP Human Development Index (HDI) – see Fig. 3 - which is a summary measure of average achievement on key dimensions of human development: a long and healthy life, being knowledgeable, and having a decent standard of living. The Inequalities-Adjusted Human Development Index (IHDI) takes account not just of a country’s achievement on health, education and income but also of how these achievements are distributed. It does this by ‘discounting’ each dimension’s average value according to its level of inequality. Under perfect equality the HDI and the IHDI would be equal but the IHDI falls below the HDI as inequality rises. The difference between the two measures is the loss to human development because of inequality. There is a strong correlation between the HDI and GNI per capita but Rwanda and Malawi have higher HDIs than would be predicted from their GNI per capita and Angola a lower one. Angola, Namibia and Botswana have a noticeably larger inequalities gap than the other countries.

The ‘inequalities’ adjustment emphasises the effect of poverty on development, but there are other ways in which development can be unequal – the differences between men and women, for example. UNDP also produces a Gender Development Index, which shows the difference in human development by gender. There are significant differences in all countries, with women having a lower index value than men, although the differences are noticeable smaller in Rwanda and Namibia than the other countries for which the data are available.

An alternative approach is to have a dashboard of indicators and compare them independently. The MDGs are one example of this. The advantage of such an approach is that we can see progress across a range of indicators and this can give guidance on the issues and areas on which attention needs particularly to be focused. However, a dashboard approach does not take account of the correlations between the different indicators and it can leave it open to policy makers to decide what it is that indicates progress when there is improvement on some but not on other indicators - it is possible to focus on some indicators and ignore or discount others. With the MDGs, for example, more attention has been focused on reducing poverty than creating decent jobs. Furthermore, it is possible to make progress towards achieving targets on many of the MDGs without tackling the exclusion of the most disadvantaged groups.

**Fig. 4:**
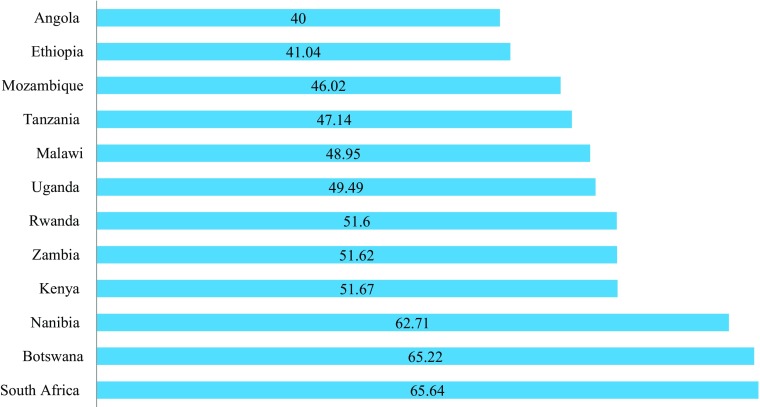
Social Progress Index 2015 for Selected East and Southern African Countries Source: Porter et al. 2015 (Note: no Social Progress Index Score is available for Zimbabwe or Comoros)

**Fig. 5:**
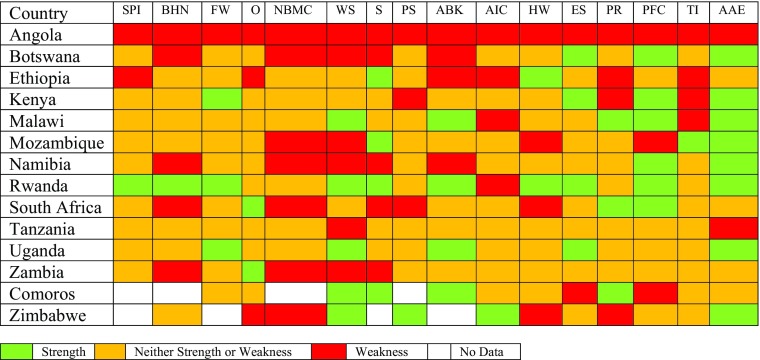
Summary Scorecard for Social Progress in Selected East and Southern African Countries - Relative Progress Compared to 15 countries with Most Similar GDP

The Social Progress Index (Porter et al. 2015 - Fig. 4) aims to combine the advantages of a single index with those of a dashboard. It provides a rich framework for measuring social progress by combining indicators into three domains - Basic Human Needs, Foundations of Wellbeing and Opportunity - and then combining the three domains into a single index. A summary scorecard (Fig. 5) is then able to show where countries have *strengths* relative to the 15 countries with the most similar GDPs, *neither strength or weaknesses*, or *weaknesses*. This provides a realistic assessment of what it is possible for a country with a given GDP to achieve and guidance as to where more investment/effort by government might be worth considering. The most interesting finding, looking at the overall index, is the low score of Angola, which has the fourth-highest GNI per capita but a noticeably lower Social Progress Index score than all the other countries. Botswana also scores lower than might be predicted from its GDP. Rwanda, Uganda and Malawi score marginally higher than might be expected.

Figure 5 provides more detail that enables us to see how the countries are doing compared with other countries that have comparable GDPs. On the overall Social Progress Index Rwanda is performing above expectation while Angola is performing below it; indeed, Angola is below expectation on every indicator. Rwanda only performs below expectation on Access to Information and Communication (access to telephones and the internet, freedom of the press) while it performs above expectation on 10 of 16 indicators. Where a country is performing below expectation (red) it suggests that governments could pay more attention to this issue and where countries are performing above expectation (green) other countries may be able to learn from their success. The Rwandan shortfall is unexpected. While internet access is still limited compared with more prosperous neighbours despite investment in cable and some public provision, mobile phones are now commonplace in towns and reasonably numerous even in the country; however, press freedom is judged to be limited in some important respects.

### Subjective Evaluations

**Fig. 6:**
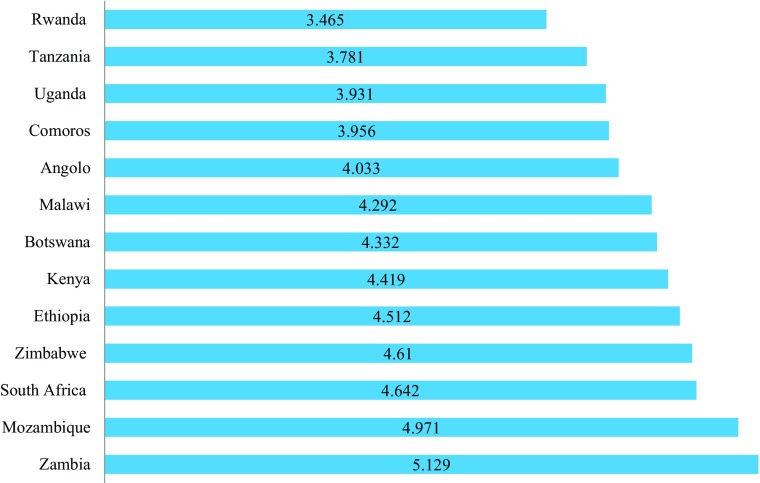
Life Evaluation Index 2012–2014 in Selected Countries in East and Southern Africa (Source: Helliwell et al. 2014) (Note: no score is given for Namibia)

In recent years governments have begun to acknowledge the importance of taking account of citizen’s own evaluations of their lives. Subjective satisfaction has been shown to be a relatively stable cognitive evaluation of an individual’s life situation that is influenced by more than economic circumstances. Helliwell et al. (2014), for example, show that variations between countries in life evaluation are explained by GDP per capital, social support, healthy life expectancy, freedom to make life choices, generosity and perceptions of corruption and how the relative importance of these factors varies by country. Figure 6 shows average scores out of ten, and life satisfaction is relatively low in all the countries in East and Sothern Africa. The highest ranked country in the world is Switzerland with a score of 7.5, the highest in sub-Saharan Africa is Zambia (5.1) and the lowest in Sub-Saharan Africa are Burundi at 2.9 and Togo (the lowest score in the world) at 2.8. Interestingly, Zambia and Mozambique, two of the poorest countries, with the highest levels of poverty and, in the case of Mozambique, the lowest HDI and next-to-lowest Social Progress Index score, have the highest average ratings. Zimbabwe also has a higher rating than might have been expected from the more objective measures, as does Ethiopia, while Botswana has a lower one.

Societal progress is about improvements in the well-being of people and households. Assessing it requires looking not only at the functioning of the economic system but also at the diverse experiences and living conditions of people. The OECD Framework for Measuring Well-Being and Progress is based on the recommendations made in 2009 by the Commission on the Measurement of Economic Performance and Social Progress to which the OECD contributed significantly. It also reflects earlier OECD work and various national initiatives in the field. This Framework is built around three distinct domains: material conditions, quality of life and sustainability, each with their relevant dimensions. As developed in the ‘Better Life’ Index, the Index obtains subjective evaluations on 13 dimensions, from housing, income and jobs to political engagement, life satisfaction and work-life balance, weighted by the extent to which the participants themselves regard them as important (OECD 2016a, b).

## Social Quality and the Decent Society Model

**Fig. 7:**
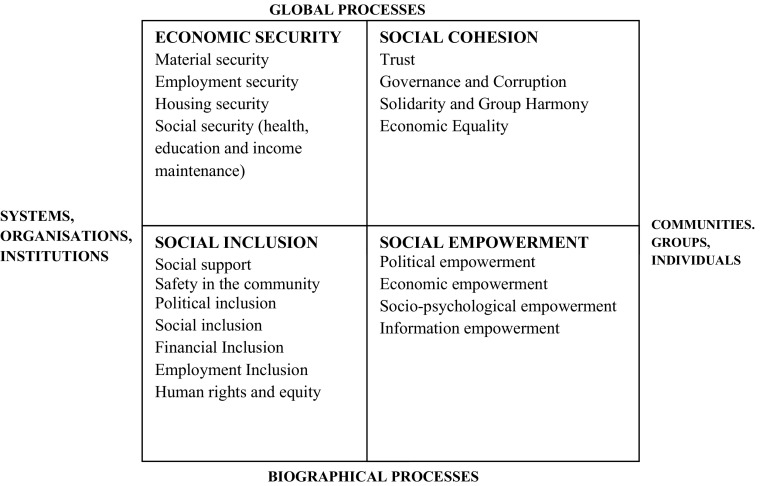
The Decent Society Model

The measures of social progress that we have reviewed so far do not give us a very clear picture of East and Southern Africa and do not provide clear and unambiguous guidance on policy priorities. (The one clear message that comes across is that there are socio-economic problems and inequalities.) However, another way of modelling what is going on may be more useful. The Social Quality approach was originally developed as a counter to what were perceived at the time as the overly economistic policies of the European Union (see e.g. Beck et al. 1997, van der Maesen and Walker 2012) and has been further developed by Abbott et al. (e.g. 2016) into a model of social provision rather than social experience – the Decent Society Model. Sociologically informed, the Model identifies four different kinds of social process with which a society has to engage successfully in order to be counted as decent and sustainable (Fig. 7; Abbott et al. 2015, 2016). The four dimensions are Economic Security, Social Cohesion, Social Integration and Empowerment. The first two measure the quality of the society at the level of system integration, whilst the other two measure how individuals are integrated into the societal structure. Governments need to develop explicit policies to address all four quadrants. A sustainable society needs to be cohesive and inclusive, to build the capabilities of its members and to provide economic security for all its citizens. Analytically we can separate these four elements, but they are inextricably interconnected, with each providing the conditions for the others and together creating a whole that is not reducible to its elements.

The Model aims to provide a robust and holistic measurement framework that can be used by governments and citizens to identify areas where policies and programmes need to be developed and implemented to accelerate sustainable inclusive development. Rather than judging progress, it provides a theoretically informed framework that enables us to ask whether a society

- has the basic building blocks in place to improve the quality of life of all its citizens and enable them to lead a life they value, and
- has the foundations for building a just society (Fraser 2010) - a society where there is parity of participation.

It sees the social as central to quality of life and challenges the subordination of social welfare and welfare policies to the economy and economic policies. It is structural and has a societal perspective with strong theoretical and ontological foundations; it shows recognition of the interdependency of human beings and the ‘conditional’ or ‘foundational’ components of their experiences, including opportunities and contingencies. It provides a framework for thinking about the goals of social development and a theorised basis for measuring progress, and it is also a policy instrument, acting as a guide to how societies can organise themselves to provide decent living conditions. Again, there is no society to which we can point and say ‘that one is the decent society’, and we have deliberately left the concept loosely defined, but its meaning is understood and it does provide a principle in accordance with which many governments frame their policies or at least wish to be seen as doing so.

*Economic Security* is about more than not living in poverty: it is about a society being able to ensure that citizens have confidence that they will have sufficient material resources in the future as well as in the present. The central focus of economic security is managing risk and the creation of life chances; it is about ensuring that strategies are in place to enable individuals and households to cope with, for example, ill health, old age or adverse climatic conditions without being propelled into poverty. All citizens irrespective of class, gender, ethnicity, age, disability, sexual orientation or other differentiating characteristics are equally entitled to economic security. Material security is necessary for social action: if all of life has to be spent looking for food, clothing, shelter and fuel, there is little time or energy left for undertaking anything else. It does not stand alone, however; Empowerment, for example, affects the working of the economy at both household and national level, and adequacy of material resource is also an aspect of social inclusion. The creation of life chances is about the provision of tools and knowledge, the essential resources to enable individuals and communities to support themselves. Critical here are education and health services as well as employment services and opportunities. The Model does not stipulate how social welfare is to be provided; different societies are at different stages of development, and for the poorest nations the extended family or immediate community may well be the main source of welfare. However, governments have a duty to ensure that the conditions are in place to enable families and communities to provide mutual support and to lift the poorest sustainably out of poverty.

*Social Cohesion* is the extent to which individuals and groups of people share social relations - the extent to which groups whose interests may not coincide can tolerate each other and live with diversity (O’Connor 1998). It is the process which integrates different groups and interests at a structural level and manages conflicts and cleavages. Building social cohesion is essential for a sustainable society; it is the basis of social order and provides the foundation for economic development and growth. Underpinning social cohesion is trust in the generalised other and confidence in government and civic and private-sector institutions. Trust provides the basis for social interaction and enables people to work together to achieve common ends. People or groups may have different goals and hold different values, but for there to be a society which functions and is sustainable there must at least be an agreement on (a) how disputes and clashes of interest are mediated, (b) how and to what extent ‘destructive’ behaviours are controlled and (c) how the minority is protected from the majority and both are protected from the power of the state. In other words, a shared expectation (preferably taken for granted) is the foundation on which the acceptance of mutual obligations and reciprocity are built, enabling self-interest to be replaced by a commitment to promoting collective interest, in both the political and the commercial spheres.

*Social Inclusion* is membership of a society – citizenship as the basis for participating in the social, economic, political and cultural institutions of a society (Levitas 1998). This requires:

- the dismantling of institutional obstacles to participatory parity, whether economic, social or political;
- ensuring that people have sufficient economic resources to participate in the taken-for-granted activities and are recognised as fundamentally equal members of society; and
- that everyone’s interests are represented in the decision-making process over redistribution and recognition (Fraser 2010).

It is based on the recognition of rights and responsibilities, accountability and judgement and of the fundamental equality of all, and on the provision of life chances for all members of society to participate in the activities of society (Abrams et al. 2004; Sen 2000). It enables individuals to claim and exercise their human rights and use their capabilities to achieve goals (objective wellbeing) through society’s opportunity structures (Sen 1999). Performance (participation in institutions) develops a shared understanding of values and behavioural expectations and it engenders interpersonal trust and solidarity, loyalty and social cohesion. Social inclusion is about more than money, although lack of material resources is one form of social exclusion (Bowring 2000; Giddens 1994). There are three levels of inclusion: the micro (interpersonal integration in close-knit/informal networks of family, friends and neighbours), the meso (civic integration through membership of formal organisations which build trust, shared norms, solidarity and loyalty and permit coordinated action), and the macro (social, economic and political integration through citizenship rights).

*Empowerment* is the provision of what is necessary for people to exercise agency and act autonomously; it increases the range of human choices by building people’s capabilities to achieve the better life they desire. Empowerment means that people have both the freedom and the capabilities to act in whatever way they deem important (Sen 1999), with the proviso that others are entitled to precisely the same rights. It is about both building capabilities and providing the conditions for people to be able to exercise their capabilities once built. Education, for example, builds capability for employment, but for people to be able to exercise their employability capabilities, employment opportunities have to be available. This is dependent on economic policies and requires that policy makers have decency in mind as an aim of policy.

These four ‘quadrants’ form a whole, but they are not simply additive; each of the four provides the basis for the others in complex and varied ways (Herrmann et al. 2012). Social Inclusion and Empowerment are essential for the operation of a Decent Society, while Economic Security and Social Cohesion provide the structural base. Empowerment is essential if people are to act and for them to be socially included (Sen 2000), while Social Inclusion, through the building of social capital, supports the development of Social Cohesion (Abbott et al. 2014). Economic Security provides the material resources that support Empowerment and Social Inclusion. Social Cohesion, through contributing to sustainable economic development and functioning social institutions, provides an essential foundation for Economic Security (Dulal and Foa 2011; Hamilton and Ruta 2006; Knack and Keefer 1997).

## Methods of Research

The indicators on which the Decent Society ultimately rests come mostly from the main international collations of statistical data - the World Bank Development Indicators and the UN’s statistics. It also employs databases held by specialist organisations such as Transparency International, Fund for World Peace and the findings from the Gallup World Poll and the World Values Survey. (The reader should consult Abbott et al. (2016) for details of the Index’s construction and more detailed consideration of debates around the theoretical underpinnings than can be covered here.) Some indicators that would have been valuable - for example, the proportion of the adult population that is married - were not available on any of the international databases that were consulted. For others they were not available for sufficient countries to allow a global picture to be developed (as any country which has missing data on any of the Index’s constituent variables cannot be scored and drops out of the analysis). Search engines were used to identify academic research, research reports emanating from major international NGOs and world agencies, government speeches and internal sources such as budget statements or even the occasional newspaper report to permit a few gaps on some variables for individual countries to be filled. In the event, data were collected for 121 countries.

Where information entails a subjective judgment (e.g. attitude/belief items) or where it is difficult to collect with precision (‘percentage of population undernourished’ would be an example) or where more than one indicator ‘naturally’ occurs (e.g. public health provision – clean water and safe sewage disposal) two or more measures were averaged if possible to get a working indicator score. Original items were often not measured on the same scale (e.g. percentage agreement being ‘averaged’ with a mean value on a rating scale), so all were standardised with mean of 50 and standard deviation of 10 Indicators were then averaged as domain scores. For example, the ‘Social Wage’ score in Economic Security is a composite of spending on health, spending on education and, as spending on social security was not available worldwide, a rating of extent of provision in that area. Sometimes there were sub-domains; for example, ‘Health’ in Empowerment is a composite of public health provision, medical provision and achieved health status, each of which is a composite measure. The domain scores are then an averaged to provide quadrant scores, and the quadrants were averaged to provide an overall Decent Society Index score. At each of these stages the constituent scores were re-normalised to spread the standard deviation, which tends to shrink when you add correlated variables together. The arbitrary rule for the final DSI score was that the top cases should score in the 90s if possible and the bottom ones below 10 if possible but above zero, to provide a figure that is readily interpretable. The worldwide mean throughout is 50. (For the purposes of this paper, however, we have sometimes considered scores as deviations from the mean of Eastern and Southern Africa, because all the countries in this analysis score below the world mean on most or all of their quadrant scores.)

The Decent Society Index differs from others in being concerned less with current achievement than with whether the society has the *conditions* in place for a decent performance to be achieved. Mostly we are just as interested, or more, in medical and public health provision as in how healthy the population are now and just as interested in formal acceptance of international codes of human rights as in current breaches of them. On the whole, therefore, we have preferred ‘objective’ measures or expert judgments to ‘subjective’ responses to questionnaires. Where there is no alternative because no ‘objective’ measure is available worldwide for an important indicator then we have used questionnaire responses, and we have used measures of achievement where it is not possible to evaluate separately whether the *conditions* for achievement are in place, but even here we have preferred report of ‘factual circumstances’ to subjective evaluations (‘I have a condition that handicaps me’ rather than ‘I think I am as healthy as other people of my age’). The partial exception is the Empowerment quadrant, where (a) achievement may itself be a condition – a country’s achievement of population health and education is prerequisite for citizens to be empowered – and (b) ‘typical psychological state’ may be important – we have computed domains to cover the extent to which people believe they have some freedom of choice and the extent to which they think they can ‘get ahead’ by their own hard work.

The prime indicators, domains and quadrants are listed in the Appendix.

## Research Findings

**Map 1:**
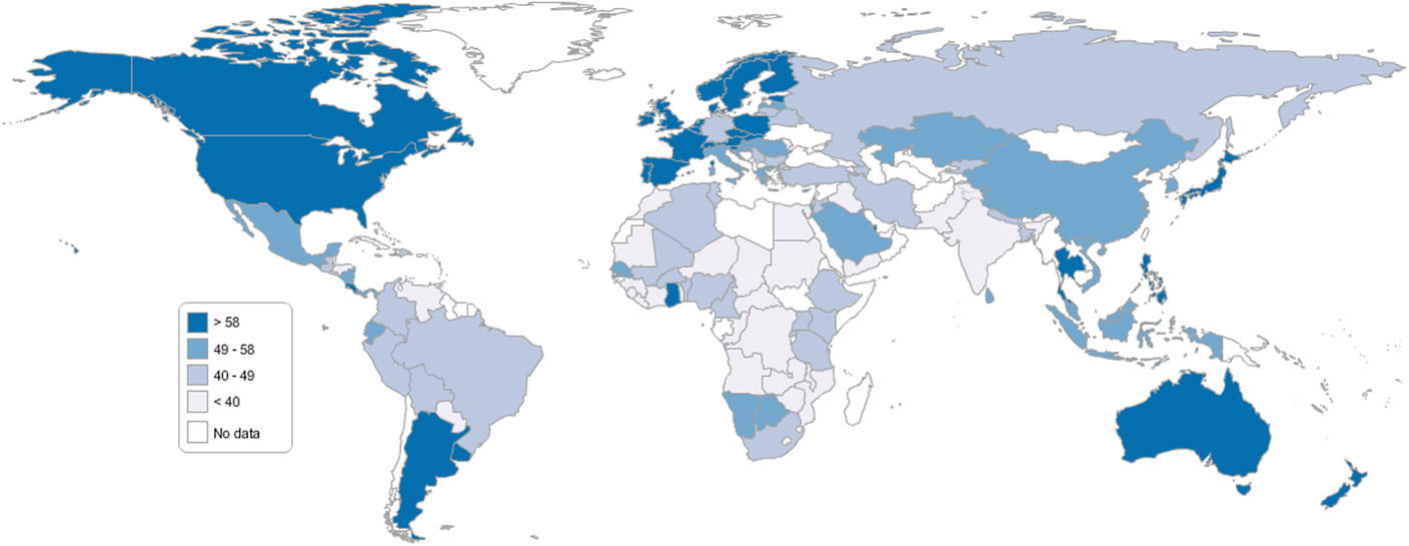
World Map: Decent Society Scores

The Decent Society Index enables us to examine the extent to which a country has the basic infrastructure to build a decent society, and there is sufficient variation in East and Southern Africa for it to be usefully applied there. The country with the highest Index in the world is Norway and the one with the lowest is Haiti. The country with the highest score in Sub-Saharan Africa is Ghana, which is ranked equal 49th in the world, and the one with the lowest score is the Democratic Republic of Congo, which is ranked 118th. In East and Southern Africa the highest-ranked country is Namibia, ranked equal 51st, and the lowest is Comoros, ranked 116th. Map 1 below divides the world into four groups.

- None of the countries of sub-Saharan Africa appear in the top group, which is filled mostly by North America, Western Europe and Australasia. In Eastern and Southern Africa,
- Namibia and Botswana are in the second group,
- South Africa, Rwanda, Uganda, Kenya, Tanzania, Ethiopia and Malawi in the third,
- and Mozambique, Zambia, Zimbabwe, Angola and Comoros fall in the bottom one.

**Fig. 8:**
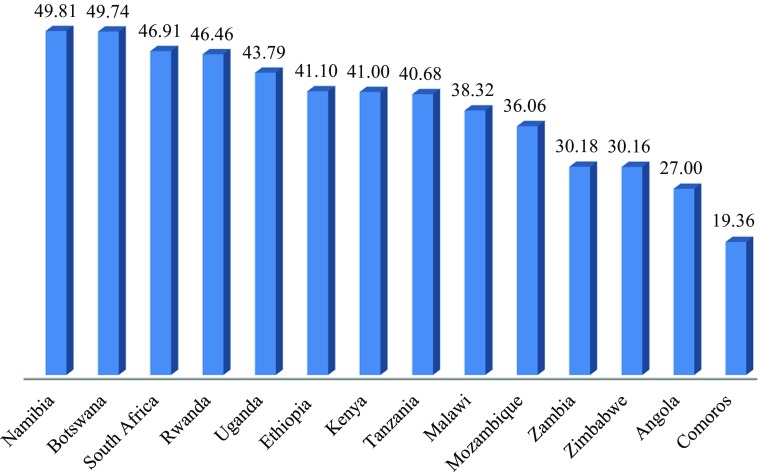
Decent Society Overall Index Scores for Selected Countries in East and Southern Africa

Figure 8 shows the scores for the fourteen countries on the overall Index. While none of the countries has all the foundations in place to build decent societies, some are clearly providing more than others. No country or countries appears to be way ahead of the rest, but Comoros has a noticeable lower score than the other countries and there is a clear gap between Zambia, Zimbabwe and Angola and the others. Although there is a relationship with GNI per capita, some countries are not doing as well as might be expected given their relative GNIs. Angola and Zambia stand out as having lower scores than would be predicted from GNI per capita and Rwanda, Uganda, Ethiopia and Malawi have higher scores than would be predicted.

**Fig. 9:**
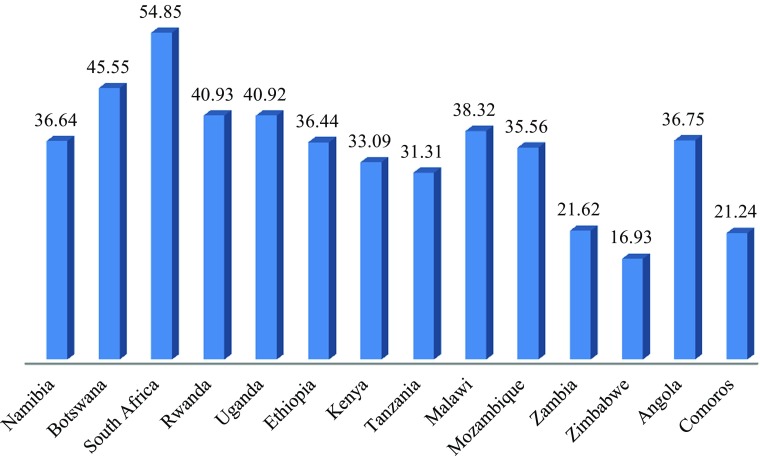
Economic Empowerment for Selected Countries in East and Southern Africa

**Fig. 10:**
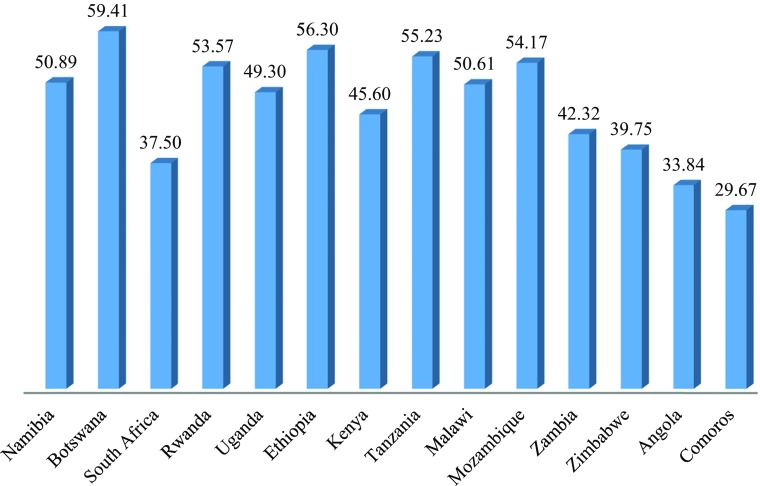
Social Cohesion for Selected Countries in East and Southern Africa

The Index is constructed from Indicators aggregated into Domains which in turn are combined to yield an overall rating or ranking, and it is possible to drill down and examine the extent to which foundations are in place for each quadrant and even to drill down further and look at domains or even individual indicators. Figure 9 shows the scores on the Economic Security quadrant. South Africa stands out as the best country, with Botswana, the country with the highest GNI, some way behind (because of the inclusion of the ‘social wage’ in the measure) and Namibia providing less economic security than some much poorer countries. However, South Africa, scores much lower on Social Cohesion (Fig. 10), and Angola and Comoros also have noticeably lower scores than the other countries. Botswana, Rwanda, Ethiopia, Tanzania and Mozambique score noticeably higher than the others.

**Fig. 11:**
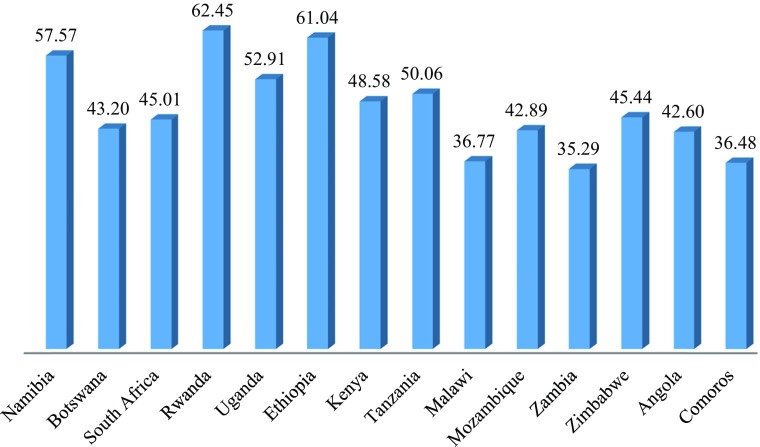
Social Inclusion for Selected countries in East and Southern Africa

**Fig. 12:**
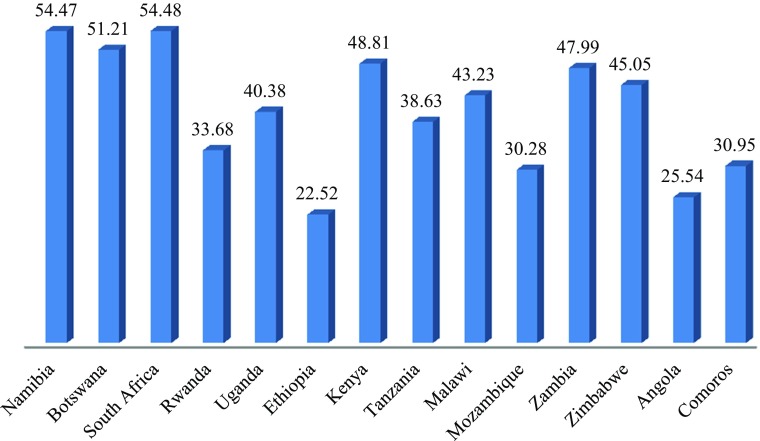
Social Empowerment for Selected Countries in East and Southern Africa

On Social Inclusion, Rwanda, Ethiopia and Namibia score noticeably higher than the other countries (Fig. 11). Malawi, Zambia and Comoros, on the other hand, score notably lower than the other countries. On Empowerment the three countries with the highest GNI per capita have noticeably higher scores than the other countries. However, Angola, the country with the fourth highest GNI per capita, scores noticeably lower than all except Ethiopia, which is the next to poorest. Malawi and Mozambique, two of the poorest countries, score higher than would be predicted from their GNIs per capita (Fig. 12).

**Fig. 13:**
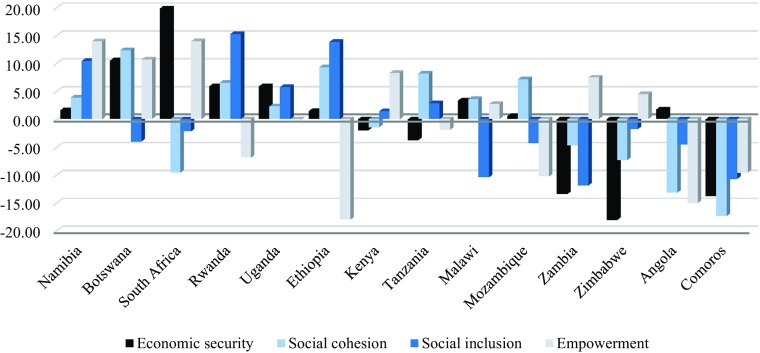
Deviation from Mean for East and Southern Africa on the Quadrants of the Decent Society Index for Selected Countries in East and Southern Africa

Figure 13 enables us to see how the countries of East and Sothern Africa are doing, compared with each other, on each of the quadrants of the Model. Namibia and Uganda are the only countries to score above the mean for East and Southern Africa on every quadrant, but Namibia scores comparatively poorly on economic security, especially given its higher than average GNI per capita, and it is also only marginally above the mean for Social Cohesion. Uganda, one of the poorer countries, scores noticeably above the mean for Economic Security and Social Inclusion but only marginally so on the other two quadrants. By contrast, Comoros scores noticeably below the mean on all four quadrants. Angola scores only marginally above the mean for Economic Security, despite being one of the more affluent countries, and noticeably below the mean on the other three quadrants. Zimbabwe has the lowest score for Economic Security and, as is the case also for Zambia, scores above the mean only for Social Empowerment. South Africa has the highest score for Economic Security and also scores comparatively well on Social Empowerment but does poorly on Social Cohesion and Social Inclusion. Ethiopia and Rwanda both score comparatively poorly on Social Empowerment but comparatively well on Social Inclusion and Social Cohesion.

Social inclusion is evidently important for decent development, looking at the quadrant scores in comparison with the overall ranking on the Index. At the bottom of the rankings on the Index, Comoros scores a fair way below the mean in all quadrants and Angola also scores predominantly below the mean although it does show some strength in terms of economic security. The next four (Zimbabwe, Zambia, Mozambique and Malawi) all show below the mean for social inclusion, though in the case of Zimbabwe this is by no means the most acute of its problems. The only other countries to score a small distance below the mean are Botswana and South Africa, near the top of the ranking, and this failure is more than compensated by strengths in other areas.

## Conclusions

Not only does socially inclusive development produce just and sustainable societies, but it also accelerates economic transformation and growth. For there to be economic progress based on the efforts of all a country’s residents, all residents must feel that they have a part in the society and that the society is fair, affords them their human rights and recognises them as in some sense citizens. Some degree of economic security is prerequisite – not much can be achieved if part of the population is struggling to survive and bring up their children on a day-to-day basis, and people need to feel safe and supported. Social cohesion is equally important – groups must be able to work together, even if their interests and ultimate goals are different. There must be agreement about ‘the rules’ and the obligations of key social roles (or else neither government nor finance and commerce can function), people must feel that the rule of law protects them and applies to all, and there must be freedom from arbitrary oppression by powerful minorities, by the majority group or by the state itself. Beyond these three, empowerment is important: people must know what is possible for them and have the will and the resources to attempt it. Citizens must know what their rights are and be able to claim and exercise them. Thus inclusion is only one of the types of process needed for the achievement of the decent society, but it is an important one.

Existing approaches to monitoring and evaluation may not be fully adequate. While it is important to be able to measure socioeconomic progress, including the extent to which it is inclusive, measures of what has been achieved do not provide guidance to countries on the legal and policy framework they need to put in place in order to provide a decent society for all citizens. Moreover, without a framework of social theory within which to site the indicators it is not easy to see what they convey about the state of the society as a whole. We have proposed an alternative, theorised approach here based on an analytic framework which has proved useful for capturing the different aims and abilities of a variety of societies, both developed and developing or in transition after economic catastrophe or civil war, and designed to give reasonably specific advice on what needs doing next to achieve a society which is more decent than before.

It should be noted, however, that there are *two* elements (or perhaps *three*) to the measurement of whether resources/infrastructure are available in a society. The first requirement is that the resource shall be assured by formal/institutional means – that a ‘policy space’ shall be created within which it can be delivered – and the second is that policies shall actually be implemented in practice. Where possible the Decent Society Index combines at least two measures within a domain or quadrant to ensure that both of these are captured. The ‘human rights’ domain, for example, consists of a rating scale based on United Nations reports (United Nations Treaty Collection) of whether countries have accepted basic Human Rights conventions combined with an assessment by Fund for Peace as part of the Fragile States Index of whether human rights are actually being breached. Health and education are measured (under Economic Security) by share of budget or GDP spent on them as part of the ‘social wage’ but also (under Empowerment) by direct measures of health and educational outcomes. The third element is fair distribution: if the society has/creates the resources, does everyone have access to them? Sometimes this can be covered by the same kind of stratagem: for example, GNI per capital (under Economic Security) is supplemented by the Gini coefficient (under Social Cohesion) and gross poverty (under Social Inclusion). Sometimes the problem cannot be solved in that way, however, and an aggregate figure at any level may be concealing gross inequalities by gender or ethnic group or age. The answer is probably a disaggregated Index, but this is not easy in a world which produces few disaggregated statistics.

What is distinctive about the Decent Society approach is that it does not sit in judgment and declare a given society to be ‘good’ or ‘failing’ and admits that different solutions may be what is needed in different countries as they strive to become decent societies, an achievement that is always in the making. It looks to see what different countries’ solutions offer from which other states may learn and where its performance against its peers suggests that improvements could be made. Rwanda’s case, for example, might suggest to others that increasing inclusiveness is ‘good politics’ and might have an economic pay-off, if such a poor country can manage to make social provision through volunteer and community labour despite a shortage of monetary resource, but the low Empowerment score brought about by restriction of political rights and freedoms might suggest that a somewhat different approach to the same goals might be preferable because such limitations on the nature of the state into which citizens are included may be too high a price to pay. It is possible, within this approach, to ‘drill down’ to quite some depth to identify precisely where intervention may be most fruitful.

The approach is not value-free – it takes for granted that governments shape cultures and assumes that what governments wish is to create a society which the western world would call ‘decent’, one where people know what is possible for them and are free (and have the resources, both economic and personal) to fulfil their own wishes and capabilities, within the constraint that their success does not constrain or disadvantage others. In our experience of talking to people in many cultures a western model of the ‘good life’ does appear to be what the people of the world want, and they will get it if they can.

## Appendix

**Table 1:** Variables in the Decent Society Index (Where a source is not attributed the data come mostly from the World Bank.)

Quadrant and domain	Prime indicator	Variables	Notes
Economic security			
National Economy	Gross national income (GNI) per capita ($ppp)		Weighted x 2
	Balance of payments (%GDP)		Negative scores on the raw indicator indicate an outflow from the economy
	Gross domestic savings (%GDP)		
	Foreign direct investment (% GDP)		
	Development aid (%GDP)		Weighted x 0.5. Polarity reversed when computing domain
	Remittances from abroad (%GDP)		Weighted x 0.5. Polarity reversed when computing domain
Food security	Food Security - average of FAO and GFSI % undernourished	% undernourished FAO (Food and Agriculture Organisation)	
		% undernourished GFSI (Global Food Security Index – Economist Intelligence Unit)	
Social Wage	Government spending on health (% of govt. expenditure)		
	Government spending on education (% GDP)		
	Social Support - Index calculated from SSA (Social Support Agency) data		Rating: Covers 5 areas: old age/disability, sickness and maternity (cash alone or cash plus medical treatment), work injury, unemployment, and family allowances. For each, 0 if not present, 1 if present or covered by another provision; +1 if not means-tested and +1 if any element is universal and dependent only on citizenship/ residence. Then overall, +1 if the claimant's contribution to old age benefit is less than half that of the employer, +2 if it is less than 25 % and +3 if it is free or less than 10 %.
Social cohesion			
Governance	Rule of Law	Rule of Law (Worldwide Governance Indicators – WWGI)	
		Rule of Law (Freedom House – FH)	
	Government Effectiveness	Government effectiveness WWGI	
		Government effectiveness FH	
		Government effectiveness (Gallup World Poll - GWP)	
	Regulatory quality - WWGI		
	Political stability, control of violence - WWGI		
	Legitimacy	Legitimacy – (Fragile States Index – FSI)	
		Fairness of elections - GWP	
	Control of corruption	Control of corruption WWGI	
		Control of corruption (Transparency International – TI)	
Trust in people	Trust in People	Trust GWP	
		Trust (World Values Survey – WVS)	
		Trust (Human Development Indicators – HDI)	
Trust in institutions	Trust in the courts	Trust in Legal Institutions GWP	
		Trust in Legal Institutions WVS	
	Trust in the Police GWP		
	Trust in the national government	Trust in National Government GWP	
		Trust in National Government WVS	
		Trust in National Government HDI	
	Trust in the military	Trust in the Military GWP	
		Trust in the Military WVS	
	Trust in banks etc.	Confidence in banks/financial institutions GWP	
		Confidence in banks/financial institutions WVS	
Economic equality	GINI coefficient		Polarity reversed as a domain score
Group Harmony	Group Grievance index (FSI)		Polarity reversed when added to domain
	This country is a good place for racial/ethnic minorities GWP		
Acceptance of immigrants	Immigrants as % of population		Polarity reversed when added to domain
	Good place for immigrants (GWP)		
Social inclusion			
Poverty	Income share held by lowest quintile		
	% at or below $1.25 ppp per day		Polarity reversed when added to domain
Financial inclusion	Has a bank account (%)		
Work inclusion	Employment - % aged 15+ in labour force		
	Unemployment - % of labour force unemployed		Polarity reversed when added to domain
Gender inclusion	Women in Parliament (%)		
	Women in employment (% of women, as ratio to % of employed men)		
Active involvement	Volunteer activity (%)		
	Voiced an opinion to politicians/officials (GWP)		
Family and friends	Rely on family/friends (GWP)		
	Easy to make and meet friends (GWP)		
Security	Feeling of safety	Feel safe on the streets at night in my location (GWP)	
		Feel secure in neighbourhood (WVS)	
Human Rights	Support for human rights	Acceptance of UN Conventions on Human Rights (rating scale)	For each of 17 Conventions: 4 = complete acceptance, 3 = minor reservations, 2 = serious reservations, 1 = acceptance but reservations subvert the Convention
		Breach of human rights (FSI)	Polarity reversed when added to domain
Empowerment			
Political empowerment	Freedom to dispute government position	Freedom of Expression (FH)	
		Freedom of association (FH)	
		Political pluralism (FH)	
		Voice and Accountability (WGI)	
Health	Public Health	Access to improved water (% of pop)	
		Access to improved sanitation (% of pop)	
	Medical health	Medical provision - Drs. per 1000 population	
	Achieved health	Life expectancy from birth (years)	
		Presence of handicap/disease/condition which hampers normal work (GWP) – ‘no’	
Education	Mean years of schooling in the population (aged 25+)		
	Primary school completion (% of age group)		
	Adult literacy rate (15+)		
Infrastructure of communication	Electricity (% of population with access)		
	Internet users (% of population)		
	Mobile cellular subscriptions (per hundred in population)		
Availability of work	Entrepreneurship opportunities	Good place to set up a business (GWP)	
		Ease of Doing Business Index (WB)	
	Good time to find a job (GWP)		
Freedom of choice	Freedom of choice	Personal autonomy (FH)	
		Freedom to live how like (GWP)	
		Freedom of choice (WVS)	
		Freedom of choice (HDI)	
Belief in effectiveness of action	Belief in effectiveness of action	Working hard leads to success (GWP)	
		People who work hard get ahead (WVS)	
